# Supplementary material for: Impact of COPD Treatment on Survival in Patients with Advanced Non-Small Cell Lung Cancer
Source: J Clin Med. 2022 Apr 24;11(9):2391. doi: 10.3390/jcm11092391 (PMC9104207; doi:10.3390/jcm11092391)
Supplement: Supplementary file 1 [file jcm-11-02391-s001.zip › jcm-1652825-supplementary.pdf]

Supplementary Table S1. Clinical factors associated with overall survival (multivariate analysis when applying inhalation therapy instead of COPD treatment)

**Variables**	**HR**	**95% CI**	***P* Value**
Sex, men	2.56	1.56-4.21	< .001
Age, years	0.99	0.97-1.01	.236
BMI, kg/m^2	0.95	0.91-0.99	.032
FEV_1 <50% predicted	1.24	0.88-1.76	.213
Clinical stage			
III	1.00		
IV	1.93	1.43-2.60	< .001
Chemotherapy	0.43	0.30-0.62	< .001
Inhaled therapy	0.72	0.54-0.97	.029

HR, hazard ratio; CI, confidence interval; BMI, body mass index; FEV_1, forced expiratory volume in 1 second.

Supplementary Table S2. Clinical factors associated with overall survival (multivariate analysis when applying inhaled corticosteroids instead of COPD treatment)

**Variables**	**HR**	**95% CI**	***P* Value**
Sex, men	2.47	1.50-4.06	< .001
Age, years	0.99	0.97-1.01	.230
BMI, kg/m^2	0.95	0.91-0.99	.041
FEV_1 <50% predicted	1.25	0.89-1.76	.199
Clinical stage			
III	1.00		
IV	1.93	1.44-2.60	< .001
Chemotherapy	0.43	0.30-0.62	< .001
ICS	0.67	0.49-0.92	.012

HR, hazard ratio; CI, confidence interval; BMI, body mass index; FEV_1, forced expiratory volume in 1 second; ICS, inhaled corticosteroids.

Supplementary Table S3. Clinical factors associated with overall survival (multivariate analysis when applying theophylline instead of COPD treatment)

**Variables**	**HR**	**95% CI**	***P* Value**
Sex, men	2.47	1.50-4.06	< .001
Age, years	0.99	0.97-1.01	.230
BMI, kg/m^2	0.95	0.91-0.99	.041
FEV_1 <50% predicted	1.25	0.89-1.76	.199
Clinical stage			
III	1.00		
IV	1.93	1.44-2.60	< .001
Chemotherapy	0.43	0.30-0.62	< .001
Theophylline	0.79	0.58-1.08	.135

HR, hazard ratio; CI, confidence interval; BMI, body mass index; FEV_1, forced expiratory volume in 1 second.

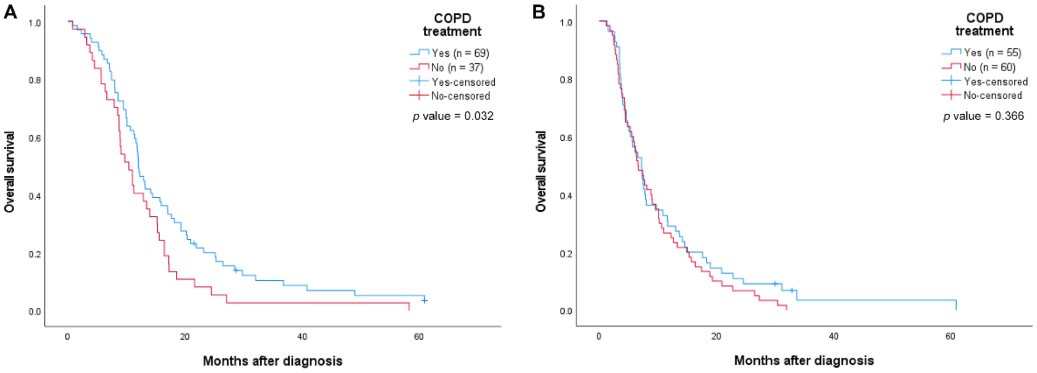

Supplementary Figure S1. Overall survival curves stratified by COPD treatment in subgroup analysis according to clinical stages; stage III (A) and IV (B).
